# Work Ability and Quality of Life in Patients with Work-Related Musculoskeletal Disorders

**DOI:** 10.3390/ijerph17093310

**Published:** 2020-05-09

**Authors:** Yi-Fang Chang, Chang-Ming Yeh, Shu-Ling Huang, Chi-Chung Ho, Ren-Hau Li, Wei-Hsun Wang, Feng-Cheng Tang

**Affiliations:** 1 Department of Physical Medicine and Rehabilitation, Han Chung Hospital, Taichung 420, Taiwan; nancy-1.04@hotmail.com; 2 Department of Occupational Medicine, Buddhist Dalin Tzu Chi Hospital, Chiayi 622, Taiwan; dm365514@tzuchi.com.tw; 3 Department of Psychology, Chung Shan Medical University, Taichung 402, Taiwan; shuling@csmu.edu.tw (S.-L.H.); davidrhlee@yahoo.com.tw (R.-H.L.); 4 Room of Clinical Psychology, Chung Shan Medical University Hospital, Taichung 402, Taiwan; 5 Department of Rehabilitation Medicine, Chung Shan Medical University Hospital, Taichung 402, Taiwan; hoc@teamtc.tw; 6 Department of Orthopedic, Changhua Christian Hospital, Changhua 500, Taiwan; cmch10011@gmail.com; 7 Department of Medical Imaging and Radiology, Shu-Zen Junior College of Medicine and Management, Kaohsiung 821, Taiwan; 8 Department of Occupational Medicine, Changhua Christian Hospital, Changhua 500, Taiwan; 9 Department of Leisure Services Management, Chaoyang University of Technology, Taichung 413, Taiwan; 10 School of Medicine, Chung Shan Medical University, Taichung 402, Taiwan

**Keywords:** work ability, quality of life, musculoskeletal disorders

## Abstract

This study aimed to investigate level of work ability and quality of life (QOL) as well as the relationship between them among patients suffering from work-related musculoskeletal disorders (WMSDs) in Taiwan. A cross-sectional study design with continuous sampling and a questionnaire were used to obtain the research data. Controlling for personal characteristics, pain, psychological distress, and social support, multiple linear regressions were adopted to explore the relationship between work ability and overall QOL. Further analyses were also made to clarify the relationships between work ability and each domain of QOL. In total, 165 patients with WMSDs were recruited. Compared with general workers, the participants reported a lower level of work ability and overall QOL. Work ability was significantly associated with overall QOL when covariates were controlled. Among the four domains of QOL, work ability was significantly associated with both the physical and psychological domains. The conclusion was that work ability is a definite factor of QOL for patients with WMSDs; the essence of work ability may be beyond economic function or social support. Strategies to help workers with WMSDs enhance their work ability to fit their new or temporary jobs would be beneficial to their QOL.

## 1. Introduction

Work-related musculoskeletal disorders (WMSDs) comprise a group of painful disorders of the muscles, tendons, and nerves related to work activities and conditions. These work activities and conditions can significantly contribute to the development of musculoskeletal disorders, and/or can cause musculoskeletal disorders to worsen or persist for longer [1]. WMSDs are common among the current working population and may cause long-term absences, necessitate disability pensions, and result in poor quality of life (QOL) [2,3]. WMSDs are also found to be associated with high costs to employers and employees such as lost productivity, increased health care, future earnings, medical/rehabilitation costs, and so on [4]. WMSDs are the single largest category of work-related illness, representing more than a third of all registered occupational diseases in the United States, the Nordic countries, and Japan [4,5,6]. In Taiwan, WMSDs account for approximately two-thirds of the applications for occupational injury and illness insurance benefits [7]. The needs and interests of the large number of employees with WMSDs should not be overlooked.

Work ability can be considered as a broader concept of employability and well-being. The term “work ability” is complex, covering not only different objectively measurable capacities, but also many aspects such as education, knowledge, skills, experience, functional capacities, work demands, and motivation [8]. Regarding the demands of work, and taking the workers’ health status and resources into consideration, the Work Ability Index (WAI) was developed as an instrument with which to assess work ability during health examinations and in workplace surveys [9]. The WAI has been applied to various countries in both field studies and clinical practice. In an 11 year follow-up study on 6259 municipal workers, a poor WAI score was a strong predictor of future work disability [10]. In another cohort study, WAI was a prominent predictor for the degree of sick leave among female employees [11]. WAI was also found to be a salient predictor of sustainable return to work in a prospective study [12]. In general, WAI score shows a significant association with QOL across sex and occupation [13,14,15]. Burnout, QOL, and work ability were also significantly interrelated categories among food-industry workers [16]. However, the studies assessing the level of work ability in the patients with WMSDs are limited.

In previous studies on occupational health, musculoskeletal discomfort such as pain was found to be associated with QOL across many industrial sectors and occupational categories. However, the definition and measure of QOL in the research varied. The intensity of musculoskeletal pain among fishing sector workers was found to be independently associated with the QOL, as measured using the 36 Item Short Form Health Survey (SF-36) [17]. A lower QOL, measured using the SF-36, was observed among artisanal fisherwomen and shellfish gatherers who had a prevalence of musculoskeletal disorders [18]. The QOL, measured using the EuroQol, was lower among sedentary office workers with low back pain than in healthy sedentary office workers [19]. The QOL, measured via common mental disorders and well-being at work, was also associated with musculoskeletal pain among teachers [20]. Additionally, even in the general population, individuals without chronic musculoskeletal pain reported a better QOL, as measured by the SF-36, than those with chronic pain [21]. By and large, individuals with musculoskeletal disorders exhibit a higher level of pain and a poorer QOL.

The WHO has defined QOL as “an individual’s perception of their position in life in the context of the culture and value systems in which they live and in relation to their goals, expectations, standards and concerns.” It is a broad-ranging concept including a person’s physical health, psychological state, personal beliefs, social relationships, and their relationships to salient features of their environment. To provide an instrument of measure that is readily available and comparable across countries, the World Health Organization Quality of Life Scale Brief Version (WHOQOL-BREF) was developed, facilitating the assessment of four domains: physical, psychological, social, and environmental [22]. Another instrument, the SF-36, has also been broadly used in research to measure QOL. In the Taiwanese population, the evidence of various studies shows that the SF-36 scale is more suitable for measuring health-related QOL for patients with chronic diseases, such as hypertension, diabetes, and stroke, while the WHOQOL-BREF is more suitable for measuring global QOL [23]. In addition, the WHOQOL-BREF domains show fewer floor or ceiling effects than the SF-36 scale does [24]. The present study therefore adopted the WHOQOL-BREF to measure global QOL for patients with WMSDs. Few studies have focused on QOL, measured by the WHOQOL-BREF, in workers with WMSDs. In these limited studies, a significantly negative relationship between WMSDs and QOL was found among physical therapists [25]. In line with this, WMSDs were negatively associated with QOL in nurses, dockworkers, and manufacturing workers [26,27,28]. However, in these studies, discriminating whether the workers had WMSDs depended on the participants’ self-reported symptoms. To ensure the work-relatedness, only patients with WMSDs diagnosed by occupational physicians were recruited for the present study.

The present study aimed to explore work ability, QOL, and their relationship among patients with WMSDs. Since QOL is affected by many factors, patients with WMSDs may suffer from pain, greater medical expenditure, sick leave, less income, and poor mental health. The related biopsychosocial factors are therefore noted in the present study. In addition to pain, psychological distress, and social support, economic status, occupational category, lesion site of WMSDs, and comorbidity were also taken into consideration.

## 2. Materials and Methods

### 2.1. Participants and Recruitment Method

The present study was conducted from July 2010 to December 2014 in the outpatient clinic of the occupational medicine department of a medical center in Taiwan, using a cross-sectional research method with continuous sampling. In different studies, the foundations for defining WMSDs were varied, including clinical pathology, presenting symptoms, and self-reported data [1]. To ensure work-relatedness, only patients with WMSDs, as diagnosed by occupational physicians, were recruited in the present study. In Taiwan, in order to claim labor insurance benefits, workers with suspected occupational diseases need to pay visits to occupational physicians, who conduct a comprehensive assessment before issuing a medical certificate of occupational disease. The diagnoses of WMSDs where the diagnostic criteria were fulfilled according to the European guidelines from the European Union Information Agency for Occupational Safety and Health [29]. The key criteria were as follows: (1) The clinical features must fit in with what is known about the health effects following exposure to the specified agent. (2) There must be indication of sufficient occupational exposure. (3) The time interval between exposure and effect must be consistent with what is known about the natural history and progress of the disease. (4) Differential diagnoses must be considered. Patients 20 years of age or older with a diagnosis of WMSDs were enrolled in the study. Lesion sites of WMSDs among the participants were obtained and grouped into three categories: upper back, lower back/sciatica, and extremities. A consent form was distributed with the survey questionnaire. To ensure the credibility of the results, the data on the questionnaire were obtained via interviews conducted by trained assistants. The study, conducted according to the Declaration of Helsinki, was approved by the Institutional Review Board of the Changhua Christian Hospital in Taiwan (CCHIRB No: 100701).

### 2.2. Measures

The survey questionnaire comprised four parts: personal characteristics, biopsychosocial factors, work ability, and quality of life. Personal characteristics included sex, age, marital status, education, occupation, and economic status. Occupation was divided into two categories: white-collar workers (including managers, professionals, technicians, office workers, and service workers) and blue-collar workers (including crafts workers and machine operators) [30]. Using a 5 point Likert response format ranging from 1 “very poor” to 5 “excellent,” economic status was measured with one question: “How would you rate your economic status at the present time?”

Biopsychosocial factors included the extent of pain, psychological distress, and social support. Pain was measured using the Dallas Pain Questionnaire, developed to assess the amount of chronic pain that affects individuals’ daily activities, work–leisure activities, anxiety–depression, and social interest [31]. There were 16 items in total in the scale; each item was rated by the participants using a visual analog scale ranging from 0% with words such as “no pain” or “not at all” at one end of a line to 100% with words such as “all the time” at the other end. Each item was divided into several segments; each segment was assigned a specific value. The sum of the values from 0 to 100 was calculated, adjusted, and transformed. This scale demonstrated strong qualities (content and construct validity, feasibility, linguistic adaptation, and international use) as an instrument to assess the severity of pain in the working population [32]. Higher scores indicated a greater impact of pain on the individual’s life. For this study, the alpha reliability was 0.90.

Psychological distress was measured using the Chinese Health Questionnaire (CHQ-12) [33]. The CHQ-12 was adapted from the General Health Questionnaire [34], with culturally relevant modifications. The twelve items in the questionnaire, including questions pertaining to depression, anxiety, sleep disturbance, somatic concerns, and interpersonal difficulties, were based on a 4 point Likert scale. A higher score indicated a higher level of psychological distress (ranging 0–36). For this study, the alpha reliability was 0.83.

Social support was assessed using the Perceived Social Support Scale, a validated instrument [35]. This scale assesses social support from family and friends, with 20 items each. A total score was obtained to represent the extent of social support (ranging 0–40). A higher score indicated having better social support. For this study, the alpha reliability was 0.92.

Work ability was measured using the Work Ability Index (WAI) [9]. The index was determined on the basis of the answers to a series of questions that considered the demands of work, and the worker’s health status and resources. This scale includes seven sections of self-assessment: current ability, work ability in relation to physical and mental demands of the job, reported diagnosed diseases and comorbidities, estimated impairment due to health status, sick leave over the last 12 months, self-prognosis of work ability in the 2 years to come, and the mental resources of the individual. The WAI is a summary measure of seven items (ranging from 7 to 49). The score indicates to what extent an employee, with respect to his/her personal requirements and existing working conditions, was able to perform his/her work. The WAI score can also be classified into four levels: poor (7–27), moderate (28–36), good (37–43), and excellent (44–49). The Chinese version of the WAI showed good internal and retest reliability and construct validity [36]. For this study, the alpha reliability was 0.68.

The QOL was assessed using the WHOQOL-BREF Taiwan Version, which was adapted from the WHOQOL-BREF for cultural specificity [22]. The WHOQOL-BREF Taiwan Version was composed of 26 questions, including physical, psychological, social, and environmental domains [37]. The response options ranged from 1 (very dissatisfied/very poor) to 5 (very satisfied/very good). The final scores of overall QOL (ranging 16–80) and of each domain (ranging 4–20) were calculated by the syntax according to the manual [38]. A higher score indicated a better perception of life quality. The WHOQOL-BREF Taiwan Version showed reliability and validity for Taiwanese individuals. For this study, the alpha reliability was 0.88.

### 2.3. Statistical Analysis

The demographic characteristics of the participants, along with their data pertaining to lesion site of WMSDs, comorbidity, pain, psychological distress, social support, work ability, and overall QOL and its four domains, were summarized using descriptive statistics. The t-test or F-test were adopted to compare the differences in overall QOL by demographic characteristics, lesion site of WMSDs, comorbidity, and level of work ability. The demographic characteristics, lesion site of WMSDs, and comorbidity showing significant differences in overall QOL were controlled for in multiple linear regressions. Pearson’s correlation was used to analyze the relationships between the continuous variables. With sex, marital status, economic status, pain, psychological distress, and social support controlled, multiple linear regression analysis was adopted to investigate the relationship between work ability and QOL, incorporating overall QOL and its four domains. The categorical covariates, including sex, marital status, and economic status, were transformed into dummy variables beforehand. Female, not married, and poor economic status were coded as referents. All statistical procedures were performed using SPSS-13.0 software for Microsoft Windows (IBM Corp., Armonk, NY, USA); a *p*-value less than 0.05 was considered statistically significant.

## 3. Results

To ensure the credibility of the results, all the data were obtained via interviews by trained assistants. In total, 180 patients were diagnosed with WMSDs by occupational physicians during the study period. Among them, 165 participants aged 27 to 68 voluntarily took part in the study. The main job titles of the participants were construction laborer, driver, cook, hairdresser, and process worker. Table 1 lists the demographic characteristics, lesion site of WMSDs, comorbidity, pain, psychological distress, social support, work ability, and QOL in the participants by sex. Of the participants, the majority were aged 41–60 (71.5%), married (83.0%), and blue-collar workers (97.6%). About half of the participants perceived their economic status as ordinary (49.4%). The lesion sites of WMSDs were upper back (18.2%), lower back/sciatica (44.8%), and extremities (37.0%). Fifty-three patients reported the presence of comorbidity. Among the participants, 17.0% had cardiovascular disease, 9.7% digestive disease, and 12.1% other diseases. The range of work ability in the participants was 11 to 38, with a mean of 22.3 and SD of 5.8. The majority of the participants reported a poor level of work ability (83.6%), and none had an excellent level of work ability.

Table 2 summarizes the comparisons of overall QOL by demographic characteristics, lesion site of WMSDs, comorbidity, and level of work ability. Sex, marital status, occupation, economic status, and level of work ability were associated with significant differences in overall QOL. Female, single, poor economic status, and poorer work ability were associated with a lower level of overall QOL. Other variables did not show any statistically significant difference in overall QOL.

Table 3 lists the crude correlations of the research variables. Except for social support in relation to pain and work ability, the other research variables, including pain, psychological distress, work ability, and overall QOL, were significantly associated with one another. Pain and psychological distress were significantly negatively correlated with overall QOL; social support and work ability were significantly positively correlated with overall QOL.

Table 4 summarizes the multiple linear regressions predicting overall QOL and its four domains. For overall QOL, with demographic factors, pain, psychological distress, and social support controlled, the model was significant (F = 28.739, *p* < 0.001; adjusted R^2^ = 0.580); work ability showed a significantly positive relation with overall QOL (β = 0.21, *p* < 0.01). For the physical domain, with the same covariates controlled (F = 27.684, *p* < 0.001; adjusted R^2^ = 0.570), work ability was positively associated with this domain (β = 0.29, *p* < 0.001). For the psychological domain, with covariates controlled (F = 16.927, *p* < 0.001; adjusted R^2^ = 0.442), work ability was positively associated with this domain (β = 0.24, *p* < 0.01). For the social domain, with covariates controlled (F = 9.218, *p* < 0.001; adjusted R^2^ = 0.290), work ability did not show a significant association with this domain (β = 0.08, *p* > 0.05). For the environmental domain, with covariates controlled (F = 8.174, *p* < 0.001; adjusted R^2^ = 0.263), work ability was not significantly associated with this domain.

## 4. Discussion

The purpose of the present study was to investigate level of work ability, QOL, and the relationship between them among patients with WMSDs. The results showed that the participants had a lower level of work ability than general workers. The participants also reported a low to moderate level of overall QOL and the worst scores in the physical and psychological domains. WAI was significantly positively associated with QOL when sex, marital status, economic status, the extent of pain, psychological distress, and social support were controlled. Further analyses were made to clarify the relationships between work ability and each domain of QOL. Among them, the relationships between work ability and both the physical and psychological domains showed significant associations. The participants reporting better work ability had higher scores in the physical and the psychological domains of QOL. In addition, psychological distress was the only variable that showed significant associations with overall QOL and all its domains.

Compared to the general population or general workers in Taiwan, the participants in the present study reported a lower score in overall QOL (mean = 51.1), particularly in the physical domain (mean = 11.3) and the psychological domain (mean = 12.7). The National Health Interview Survey showed that the overall QOL was 56.1 in the general population in Taiwan, with 15.1 for the physical domain, 13.6 for the psychological domain, 14.1 for the social domain, and 13.3 for the environmental domain [39]. Another result from a previous study of general Taiwanese workers showed that their mean of overall QOL was 59.0, with 17.2 for the physical domain, 14.1 for the psychological domain, 14.2 for the social domain, and 13.6 for the environmental domain [40]. This implied that the workers with WMSDs suffered from comparatively lower overall QOL. The main reason for this lower overall QOL was that the participants had low scores in the physical and psychological domains. These scores in the patients with WMSDs were similar to those in the patients with chronic kidney disease [41,42]. Moreover, these scores were even lower than those in the patients with diabetes mellitus in Taiwan, i.e., 53.7 for overall QOL, 13.9 for the physical domain, and 12.8 for the psychological domain [43]. It is suggested that more attention should be paid to the QOL, particularly in the physical and psychological domains, of patients with WMSDs.

Previous studies showed that the WAI score was positively correlated with overall QOL and all four domains in clinical nurses [13,44]. Those studies pertained to two premises: analyzing without the biopsychosocial factors being controlled and participants having higher scores in WAI; that is 38.4 ± 4.4 for Taiwanese nurses and 38.3 ± 6.1 for the Croatian samples. In the present study, we also found that WAI was significantly related to overall QOL, and to the physical and psychological domains as well, when covariates were controlled. The distinctive results of significance in only these two domains of QOL may come from the patients with WMSDs who had lower scores in WAI, these being 22.3 ± 5.8. It is conceivable that the WAI scores were determined by physical status; therefore, a higher WAI score appeared to predict better QOL in the physical domain [13]. But why did work ability also show a significant relationship with the psychological domain? A randomized controlled trial showed that compared with full-time sick leave, part-time sick leave improved self-rated general health and QOL in the early stage of work disability due to musculoskeletal disorders [45]. Part-time sick leave represented an opportunity for employees utilizing their residual work capacity to remain active at work and have higher work participation. Further research is recommended to explore whether self-actualization or personal accomplishment is involved in the relationship between the WAI score and QOL. In addition, since work ability is important to the QOL of patients with WMSD, modifying the job position, decreasing the physical and mental workload, increasing autonomy, and improving the physical work environment to support patients’ staying at work and to increase work ability could be strategies to enhance their QOL.

In addition to work ability, personal characteristics and adding biopsychosocial factors explained 58% of the variance in the overall QOL in the present study. Previous research has shown convincingly that pain is an important predictor for overall QOL [46,47,48]. In the present study, pain was also significantly associated with the physical domain and the environmental domain. However, some studies have shown that pain is associated with the psychological domain rather than the physical domain [49,50]. The relationship between biological and psychological conditions was more complicated than expected. In addition, the significant association between pain and the environmental domain might result from health service utilization, but verification would require more research. Notably, among the biopsychosocial factors, psychological distress was the only variable to be significantly associated with overall QOL and all the domains in the present study. Scores in depression and anxiety negatively correlated with overall QOL and all its domains in the patients with rotator cuff disease [51]. Similar results can be found among patients with chronic kidney disease and head/neck cancer [52,53]. Moreover, social support was considered important for preventing individuals’ health implications and burden, providing useful health-related information, having greater freedom to develop their daily activities, and thus perceiving favorable conditions for QOL [54,55]. In addition to the treatment of pain and WMSDs, enhancing mental health and social support would be helpful for improving QOL among patients with WMSDs.

To determine work-relatedness and explore the association between work ability and QOL, the present study recruited WMSD patients diagnosed by occupational physicians based on the basic appraisal principles rather than depending on self-reported data. The results shed some light on the relationship of work ability with the physical and psychological domains of QOL in this population; the effects of work ability were beyond economic function and social support. However, there were some limitations to the study. First, the cross-sectional study design did not allow determination of the developmental process and the causal relationship among the research variables. Some future longitudinal studies are recommended. Secondly, most of the participants were blue-collar workers. Applying the research results to white-collar workers should be done with caution. More studies are needed to clarify the relationship between work ability and QOL for white-collar workers. Thirdly, self-selection bias inevitably arose in the present study. Our findings might not represent the condition of those who chose not to participate in the study or those who did not visit occupational physicians. This bias may restrict the inference of the results. Fourthly, some other specific factors related to QOL, such as quality of marriage, burden of housework, and number of children, were not included in the present study. Further research could gather these kinds of information to expand the understanding of QOL among workers with WMSDs.

## 5. Conclusions

In the present study, to understand the level of work ability, QOL, and their relationship among patients with WMSDs, data analysis was carried out in which biopsychosocial factors and economic status were all controlled simultaneously. The results showed that the participants had a lower work ability and QOL than general workers in Taiwan. Work ability was positively associated with overall QOL, and with physical and psychological domains as well. The development of strategies to help patients with WMSDs enhance their work ability to increase their QOL is urgently needed. In addition, among the covariates, psychological distress was a salient factor associated with overall QOL and all its domains. Health-promoting programs for improving workers’ mental health might be another route to increase their QOL.

## Figures and Tables

**Table 1:** The characteristics of the participants and the means of research variables.

Variables	Male (*n* = 96)		Female (*n* = 69)		Total (*n* = 165)	
	*n*	% ^a^	*n*	% ^a^	*n*	% ^a^
Age (years)						
20–40	17	17.7	3	4.4	20	12.1
41–60	62	64.6	56	81.2	118	71.5
61 and over	17	17.7	10	14.5	27	16.4
Educational level						
elementary school	28	29.2	32	47.1	60	36.6
junior high	25	26.0	14	20.6	39	23.8
senior high and above	43	44.8	22	32.4	65	39.6
Marital status						
single	13	13.5	15	21.7	28	17.0
married	83	86.5	54	78.3	137	83.0
Occupation						
white-collar	4	4.2	0	0.0	4	2.4
blue-collar	92	95.8	69	100.0	161	97.6
Economic status						
good	20	21.1	14	20.3	34	20.7
ordinary	47	49.5	34	49.3	81	49.4
poor	28	29.5	21	30.4	49	29.9
Lesion site of musculoskeletal disease						
upper back	18	18.8	12	17.4	30	18.2
lower back/sciatica	60	62.5	14	20.3	74	44.8
extremities	18	18.8	43	62.3	61	37.0
Comorbidity						
cardiovascular disease	16	16.7	12	17.4	28	17.0
digestive disease	12	12.5	4	5.8	16	9.7
other diseases	10	10.4	10	14.5	20	12.1
Level of work ability						
poor	80	83.3	58	84.1	138	83.6
moderate	13	13.5	11	15.9	24	14.5
good	3	3.1	0	0.0	3	1.8
excellent	0	0.0	0	0.0	0	0.0
Variables (possible range)	Mean	SD	Mean	SD	Mean	SD
Pain (0–100)	37.2	19.4	38.5	20.1	37.8	19.7
Psychological distress (0–36)	9.1	5.5	11.5	5.8	10.1	5.7
Social support (0–40)	28.8	8.6	29.6	8.9	29.2	8.7
Work ability (7–49)	22.4	6.2	22.2	5.3	22.3	5.8
Overall quality of life (16–80)	52.0	6.9	49.8	7.2	51.1	7.1
Physical domain (4–20)	11.6	2.4	11.0	2.5	11.3	2.5
Psychological domain (4–20)	13.1	2.4	12.1	2.6	12.7	2.5
Social domain (4–20)	14.0	2.0	13.5	2.2	13.8	2.1
Environmental domain (4–20)	13.4	1.7	13.2	1.7	13.3	1.7

^a^ Calculated according to a percentage of the valid count.

**Table 2:** Comparing the overall quality of life by the characteristics of the participants.

Variables	*n*	Mean	SD	t/F	*p* Value
Sex				2.05	0.04
male	96	52.0	6.9		
female	69	49.8	7.2		
Age (years)				0.08	0.92
20–40	20	51.3	6.5		
41–60	118	51.0	7.3		
61 and over	27	51.5	6.6		
Educational level				1.51	0.22
elementary school	60	50.2	6.5		
junior high	39	52.7	6.7		
senior high and above	65	50.9	7.8		
Marital status				−2.49	0.01
single	28	48.1	7.1		
married	137	51.7	6.9		
Occupation				−1.22	0.22
white-collar	4	46.8	11.2		
blue-collar	161	51.2	7.0		
Economic status				10.77	<0.0001
good	34	53.7	6.0		
ordinary	81	52.2	6.6		
poor	49	47.5	7.3		
Lesion site of musculoskeletal disease				0.90	0.41
upper back	30	52.6	5.2		
lower back/sciatica	74	50.6	7.7		
extremities	61	50.9	7.1		
Comorbidity				0.03	0.98
with	53	51.1	7.3		
without	112	51.1	7.0		
Level of work ability				10.88	<0.0001
poor	138	50.0	6.7		
moderate	24	56.0	6.7		
good	3	60.2	3.8		

**Table 3:** The correlations of research variables and the reliability for the scales.

Variables	Pain	Psychological Distress	Social Support	Work Ability
Pain	_			
Psychological distress	0.498 **	_		
Social support	−0.138	−0.405 **	_	
Work ability	−0.563 **	−0.209 **	−0.074	_
Overall quality of life	−0.595 **	−0.632 **	0.410 **	0.435 **

** *p* < 0.01.

**Table 4:** Summary of multiple linear regression predicting the quality of life and its domains.

Variables	Overall Quality of Life			Physical Domain			Psychological Domain			Social Domain			Environmental Domain		
	B	*β*		B	*β*		B	*β*		B	*β*		B	*β*	
Sex ^a^	1.09	0.08		0.32	0.07		0.59	0.12	***	0.17	0.04		0.01	0.00	
Marital status ^a^	1.38	0.07		−0.05	−0.01		0.36	0.05		0.89	0.16	*	0.18	0.04	
Economic status—ordinary ^a^	0.86	0.06		0.06	0.01		0.16	0.03		0.22	0.05		0.42	0.12	
Economic status—good ^a^	2.43	0.14	*	0.69	0.12		0.41	0.07		0.54	0.10		0.79	0.19	*
Pain	−0.10	−0.27	***	−0.05	−0.42	***	−0.01	−0.08		−0.02	−0.14		−0.02	−0.22	*
Psychological distress	−0.39	−0.32	***	−0.07	−0.17	*	−0.16	−0.37	***	−0.08	−0.23	*	−0.07	−0.22	*
Social support	0.18	0.22	***	0.03	0.10		0.06	0.22	**	0.06	0.22	**	0.03	0.17	*
Work ability	0.26	0.21	**	0.12	0.29	***	0.11	0.24	**	0.03	0.08		−0.00	−0.01	
Adjusted R^2^	0.580			0.570			0.442			0.290			0.263		
F	28.739		***	27.684		***	16.927		***	9.218		***	8.174		***

B denotes unstandardized regression coefficient; *β* denotes standardized regression coefficient. ^a^ Female, single, and poor economic status were coded as reference groups. * *p* < 0.05; ** *p* < 0.01; *** *p* < 0.001.
